# Intramuscular Pressure of Human Tibialis Anterior Muscle Reflects *in vivo* Muscular Activity

**DOI:** 10.3389/fphys.2019.00196

**Published:** 2019-03-04

**Authors:** Filiz Ateş, Brenda L. Davies, Swati Chopra, Krista Coleman-Wood, William Litchy, Kenton R. Kaufman

**Affiliations:** ^1^Motion Analysis Laboratory, Department of Orthopedic Surgery, Mayo Clinic, Rochester, MN, United States; ^2^Department of Neurology, Mayo Clinic, Rochester, MN, United States

**Keywords:** intramuscular pressure, tibialis anterior, ankle torque, compound muscle action potential (CMAP), electromechanical delay

## Abstract

Intramuscular pressure (IMP) is the fluid hydrostatic pressure generated within a muscle and reflects the mechanical forces produced by a muscle. By providing accurate quantification of interstitial fluid pressure, the measurement of IMP may be useful to detect changes in skeletal muscle function not identified with established techniques. However, the relationship between IMP and muscle activity has never been studied *in vivo* in healthy human muscles. To determine if IMP is able to evaluate electromechanical performance of muscles *in vivo*, we tested the following hypotheses on the human tibialis anterior (TA) muscle: (i) IMP increases in proportion to muscle activity as measured by electrical [Compound Muscle Action Potential (CMAP)] and mechanical (ankle torque) responses to activation by nerve stimulation and (ii) the onset delay of IMP (IMPD) is shorter than the ankle torque electromechanical delay (EMD). Twelve healthy adults [six females; mean (SD) = 28.1 (5.0) years old] were recruited. Ankle torque, TA IMP, and CMAP responses were collected during maximal stimulation of the fibular nerve at different intensity levels of electrical stimulation, and at different frequencies of supramaximal stimulation, i.e., at 2, 5, 10, and 20 Hz. The IMP response at different stimulation intensities was correlated with the CMAP amplitude (*r*^2^ = 0.94). The area of the IMP response at different stimulation intensities was also significantly correlated with the area of the CMAP (*r*^2^ = 0.93). Increasing stimulation intensity resulted in an increase of the IMP response (*P* < 0.001). Increasing stimulation frequency caused torque (*P* < 0.001) as well as the IMP (*P* < 0.001) to increase. The ankle torque EMD [median (interquartile range) = 41.8 (14.4) ms] was later than the IMPD [33.0 (23.6) ms]. These findings support the hypotheses and suggest that IMP captures active mechanical properties of muscle *in vivo* and can be used to detect muscular changes due to drugs, diseases, or aging.

## Introduction

Functional properties of skeletal muscles change due to training (Duchateau and Baudry, 2011) or aging (Janssen et al., 2002), disuse (Lamboley et al., 2016), malnutrition as well as for a spectrum of acquired and inherited myopathies (Brooke et al., 1989). Weakness and atrophy are commonly recognized manifestations of muscle disorders. Muscle weakness is related to a reduction in the number of active muscle fibers as observed in inflammatory myopathies, a reduction in the number of functioning muscle fibers as might be seen in channelopathies (Jurkat-Rott et al., 2010), and/or in the muscle disorders that affect the contractile apparatus like that seen in some toxic myopathies (Lexell, 1995; Lexell et al., 1988). Historically *in vivo* evaluation of muscle function has been limited to manual muscle testing, measurement of force production, and electromyography (EMG). Although EMG is capable of quantifying neuromuscular electrical activity, it does not provide a quantitative measurement of muscle force. In some disorders, like steroid induced myopathy, the EMG may be normal when there is a reduction in muscle force production. Further, manual muscle testing is subjective. The sensitivity of the available muscle strength tests is limited. People often complain of weakness before any objective or subjective measure can demonstrate reduced muscle function (Shahgholi et al., 2012). Even if isokinetic dynamometers are used to quantify joint torque, it does not reflect individual muscle characteristics. To better understand muscle function and weakness, the quantitative assessment of electromechanical properties of individual muscles is necessary. This will provide a more sensitive and robust measure of muscle dysfunction.

Intramuscular pressure is the fluid hydrostatic pressure generated within a muscle and directly reflects the mechanical forces produced by a muscle (Aratow et al., 1993). Previous *in situ* investigations in animal muscles show a strong relationship between IMP and the active and passive muscle tension (Davis et al., 2003; Winters et al., 2009). A fiber optic sensor measures the interstitial pressure through a diaphragm that deforms due to alterations in fluid pressure and subsequently changes the output signal. Recently, we have shown that the IMP is correlated with muscle force during isometric voluntary contractions at dorsiflexion, neutral, and plantar flexion positions of the ankle (Ates et al., 2018b). Therefore, this minimally-invasive approach is a promising method to detect the electromechanical pathophysiology of skeletal muscle. However, the relationship between IMP and muscle activity levels has never been tested *in vivo* in healthy human muscles. If validated, this approach provides a new method for measuring muscle strength and weakness.

Electromechanical delay (EMD) is the time lag between the activation of a muscle and force exertion due to the underlying electromechanical processes of excitation-contraction coupling (Norman and Komi, 1979; Nordez et al., 2009). EMD is a link between mechanical and electrophysiological properties of muscle. Therefore, it is critical to not only to characterize changes in muscle-tendon properties (e.g., tendon stiffness, muscle slack length, and activation dynamics) (Morl et al., 2012) but also to identify changes in EMD due to neuromuscular diseases where the electromechanical coupling is disrupted.

The goal of this study was to test the following hypotheses on the human tibialis anterior (TA) muscle *in vivo*: (i) IMP increases in proportion to muscle activity as measured by electrical [compound muscle action potential (CMAP)] and mechanical (ankle torque) responses to activation by nerve stimulation. (ii) The onset delay of IMP (IMPD) is shorter than ankle torque electromechanical delay (EMD).

## Materials and Methods

### Participants

Twelve young healthy adults between the ages of 21–40-years old [6 females, 6 males; mean (SD) = 28.1 (5.0) years old with 25.3 (5.9) kg/m^2^ body mass index] participated. The exclusion criteria were (i) history of nervous system disorders or musculoskeletal diseases, (ii) history of surgeries on the right lower extremity, (iii) current use of blood thinners or medications that affect muscle strength, and (iv) pregnancy. This study was carried out in accordance with the Declaration of Helsinki. The procedures were approved by the Mayo Clinic Institutional Review Board. All participants provided written informed consent in accordance with the Declaration of Helsinki.

### Experiments

**Figure 1:**
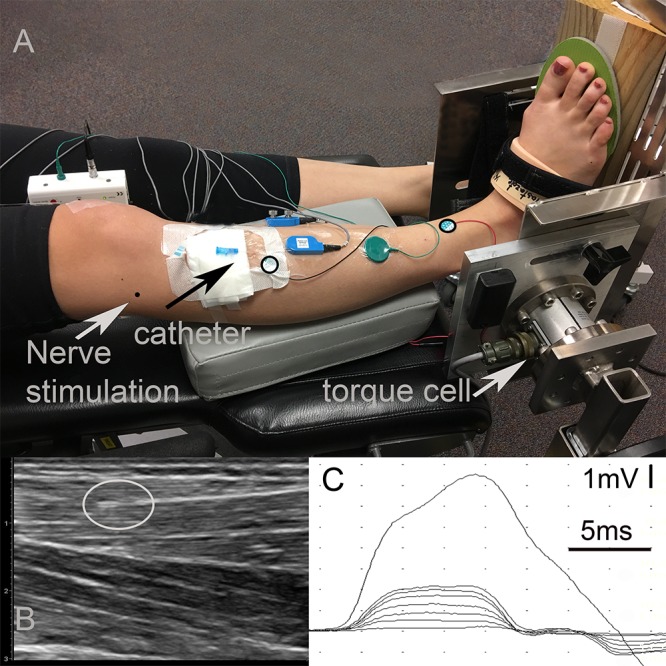
Experimental data collection. **(A)** Setup: The right foot of the participant strapped into a custom torque measurement system. The center of the torque cell was aligned with the ankle axis using a laser pointer whose light was in line with the lateral malleolus. A wooden wedge with an angle of 20° was placed underneath the foot in order to promote the optimal line of action of the tibialis anterior (TA) muscle. Black circles on the TA show the locations of the anode and cathode of the CMAP surface recording electrodes. The black arrow shows the entry of the catheter. The tip of the sensor is below the black circled electrode inside the muscle. The gray arrow shows the entry of the fine-wire needed for fibular nerve stimulation. Note that data collected from surface EMG electrodes and fine-wire EMG electrodes shown in the picture were not included into this study. **(B)** Ultrasound image of the TA identifying the location of the intramuscular pressure (IMP). The gray circle shows the tip of the IMP sensor. **(C)** Time dependent tracing of the compound muscle action potential (CMAP) for different levels of fibular nerve stimulation.

The participant was positioned supine with the foot secured to a custom torque measurement system (Figure 1A). The skin over the TA was cleaned and shaved if necessary.

### Nerve Stimulation Preparations

The fibular nerve was identified at the head of the fibula. The location was confirmed with percutaneous electrical stimulation of the nerve. A fine-wire electrode was inserted into the lateral side of knee near the nerve (Figure 1A) as the cathode for stimulation. A disposable surface electrode was placed 2 cm proximal to the cathode along the course of the nerve and served as the anode. This setup allowed for stimulation of the nerve at low currents and prevented movement of the stimulation electrodes during the experiments. All of the stimulation was performed using a Nicolet Viking EDX (Natus Neurology, Madison, WI).

### Recordings

#### Surface Electrodes for CMAP Recordings

Standard disposable electrodes (Natus Neurology, Madison, WI) with a recording surface of 15 mm diameter were used to record CMAPs. The active electrode was placed over the motor end plate of the TA, one-third the distance between the patella and the bimalleolar line, and the reference electrode was placed over the tendon of the TA at the bimalleolar line (Aquilonius et al., 1984). The ground electrode was placed between the active electrode and the stimulating cathode (Figure 1A). The CMAPS were recorded with 4800 Hz sampling frequency and were filtered on a Nicolet Viking EDX (Natus Neurology, Madison, WI) using a band pass filter of 2 Hz–10 KHz.

#### Ankle Torque

A torque cell with a max output of 565 Nm (Model 2110-5K; Honeywell International Inc., Morris Plains, NJ, United States. Non-linearity: ± 0.1% of rated output. Hysteresis: ± 0.1% of rated output. Repeatability: ± 0.05% of rated output.) was attached to an aluminum plate designed to fix the ankle angle (Figure 1A). Test-retest reliability of the torque measurement system was excellent with an intraclass correlation coefficient of 0.88 and 0.96 for plantar flexion and dorsiflexion maximum voluntary contraction (MVC). The torque cell was connected to a strain gage amplifier (SGA/A, Mantracourt Electronics Ltd., Exeter, United Kingdom) and calibrated with known weights prior to the experiments.

The ankle was immobilized at 20° of plantar flexion. The torque cell axis was aligned with the ankle axis of the participant with the help of a laser pointer whose light was aligned with the lateral malleolus. A wooden wedge with an angle of 20° was placed underneath the foot to assure an optimal TA muscle line of action (Figure 1A). The TA assists in foot inversion, so this ankle position ensured that the TA muscle line of action was perpendicular to the torque measurement system axis and, thereby, optimized its contribution to the measured joint torque.

#### IMP

For IMP measurements, a 22-gage IV catheter (Introcan Safety^®^ - B. Braun, Medical Inc., Bethlehem, PA) was inserted into the TA parallel to its muscle fibers and was positioned between the deep surface of the crural fascia and the central tendon using ultrasound guidance (ACUSON Freestyle, Siemens Medical Solutions United States, Inc., Mountain View, CA) (Figure 1A). The tip of the needle was inserted approximately two centimeters proximal to the region of the motor end plates. The stylet of the catheter was removed and a fiber optic pressure sensor (FOP-M260, FISO Technologies, Inc., Quebec, Canada) was inserted. The tip of the sensor was positioned one centimeter beyond the tip of the catheter lumen (Figure 1B). The fiber optic sensor was attached to a signal conditioning system (FPI-LS-10 Module on EVO-SD-5 Evolution Chassis, FISO Technologies, Inc., Quebec, Canada) configured for a 3000 Hz sampling frequency.

### Protocol

#### Stimulation Intensity

Using the Nicolet Viking EDX (Natus Neurology, Madison, WI), the fibular nerve was stimulated with a constant electrical current. The duration of the twitch stimulus was 0.05 ms. The current was increased as required. A supramaximal CMAP amplitude was obtained by increasing the stimulation intensity until the response did not increase and then by an additional 10% to ensure that the response was maximal. After the maximal CMAP was obtained and recorded twice, the current was reduced to the lowest level where a CMAP could be recorded. The current was then increased minimally until the smallest reproducible increase in the amplitude of CMAP response could be observed. This was repeated seven times, each time increasing the current (Figure 1C). Each stimulation level was performed twice.

**Figure 2:**
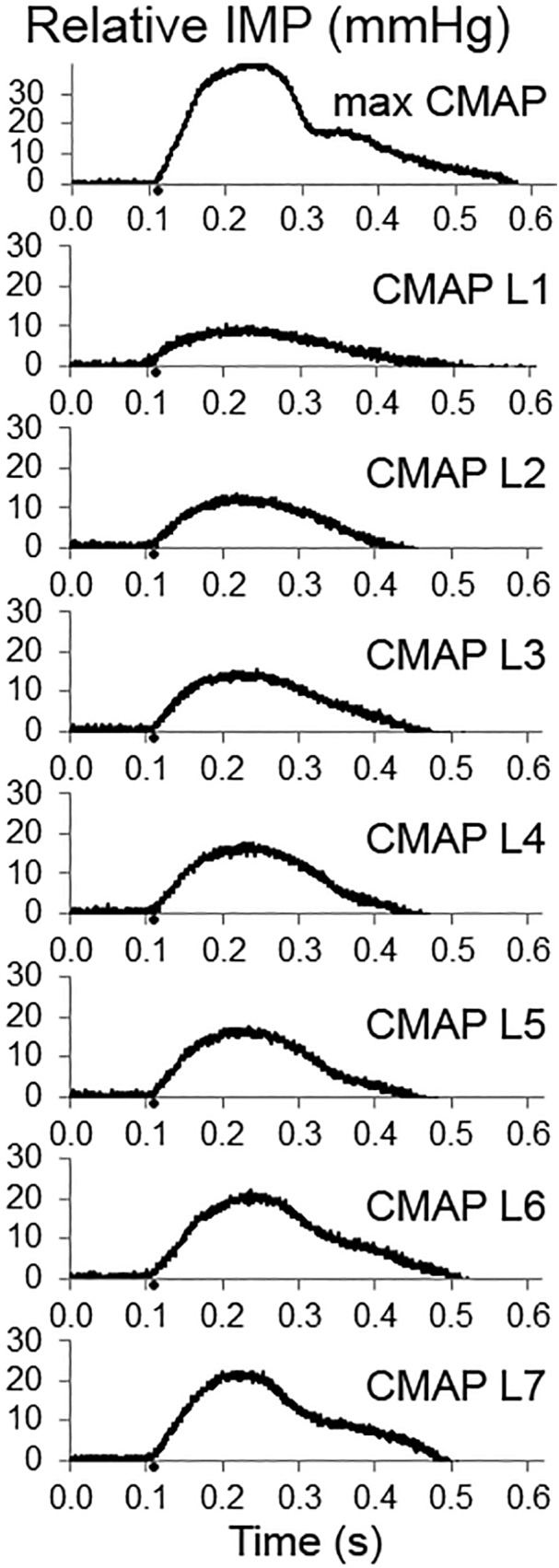
Examples of intramuscular pressure (IMP) responses to the increasing levels of fibular nerve stimulation up to obtaining a maximum compound muscle action potential (CMAP) (upper panel). The IMP responses associated with increasing CMAP amplitude are shown as CMAP level 1 (CMAP L1), CMAP L2, CMAP L3, CMAP L4, CMAP L5, CMAP L6, and CMAP L7, respectively.

The TA IMP was recorded simultaneously. Figure 2 shows an example of IMP responses to different amplitudes of stimulation: The TA IMP increases with the increasing level of stimulation.

#### Stimulation Frequency

Supramaximal stimulation of the fibular nerve was performed at frequencies of 2, 5, 10, and 20 Hz for 2 s. Each trial was repeated twice.

During all tests, the ankle torque and the TA IMP were acquired simultaneously at 3000 Hz using a 16-bit analog to digital converter (NI USB-6225, National Instruments, Austin, TX, United States) and customized software (LabVIEW National Instruments Corporation, Austin, TX, United States). To prevent muscle fatigue, a minimum of 30 s rest was provided between electrical stimulations.

### Data Processing and Analyses

Data processing was performed using custom MATLAB software (The MathWorks, Natick, MA). The baseline IMP [median 762.3 mmHg and interquartile range (IQR) 14.1 mmHg] and torque values for each trial were calculated from the mean values at rest prior to muscle activation. Raw IMP and torque data were calculated by subtracting the baseline values for each trial. Raw IMP and torque signals were filtered with a 50 Hz 4th-order low pass Butterworth filter.

#### Stimulation Intensity

The peak value of the CMAPs (Rubin and Daube, 2016) and the peak IMP responses at different stimulation intensity levels were calculated for each trial as the difference between baseline and the peak values. The amplitude and area of the CMAP were calculated on the Nicolet Viking EDX (Natus Neurology, Madison, WI). The area of the IMP response from the onset to the end, where the value equals to the onset value after reaching the peak, was calculated using custom MATLAB software (The MathWorks, Natick, MA).

#### Stimulation Frequency

**Figure 3:**
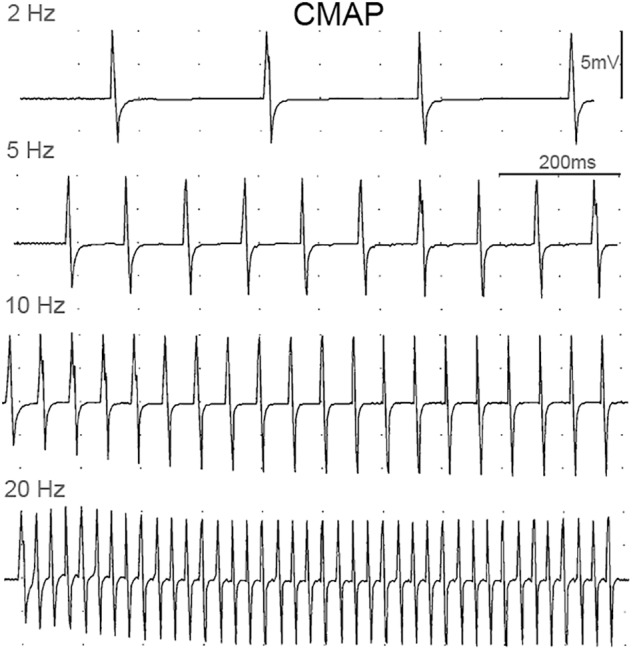
Examples of CMAP responses at different stimulation frequencies.

**Figure 4:**
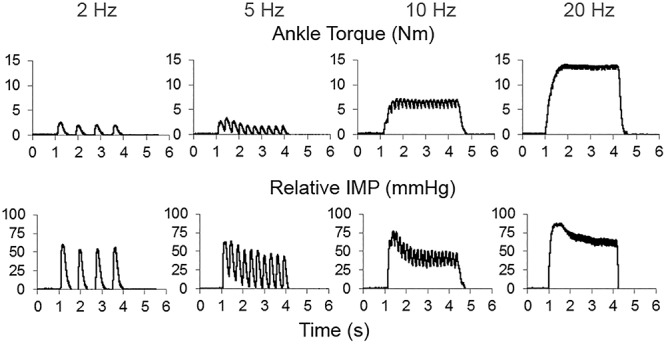
An example of time dependent tracing of the ankle torque and intramuscular pressure (IMP) at different stimulation frequencies.

The peak IMP responses, ankle torque and CMAP values at different stimulation frequencies were calculated for each trial. Figure 3 shows an example of CMAP and Figure 4 shows examples of ankle torque and IMP with respect to time for different stimulation frequencies applied.

#### Onset Delay

The onsets of CMAPs with respect to the onset of stimulation were taken from the recorded signals at Nicolet Viking EDX (Natus Neurology, Madison, WI). The onsets of ankle torque and IMP were identified using a custom algorithm (MATLAB): The quiescent value (initial 25 ms) and the mean and standard deviation of this resting state value were calculated. Signal onset was defined as the time when the signal was greater than three standard deviations above the average quiescent value (Di Fabio, 1987). Onsets were confirmed visually for each trace within each trial. The delay between the onsets of ankle torque and IMP were quantified with respect to the onset of stimulation and reported as ankle torque EMD and IMPD, respectively.

The time difference between the onset and the peak values were calculated for IMP (T_IMPpeak_) and ankle torque (T_Torquepeak_).

### Statistical Analyses

Distribution normality was tested using the Shapiro-Wilk test and statistical tests were selected accordingly. Statistical significance was set at *p <* 0.05. The Pearson’s correlation coefficient (*r*) was calculated to analyze the relationship (i) between the area of the IMP response and the area of the CMAP and (ii) between the peak IMP and the peak CMAP for different stimulation levels. A multivariate regression with each participant as a factor was performed. The *r*-squared values were calculated and the diagnostic known as *Cook’s Distance* (*Cook’s D*.) was used to detect potential influential data points. Friedman tests were used to detect (1) the differences in (i) CMAP and (ii) IMP response for different levels of stimulation and (2) the differences in (iii) ankle torque, (iv) CMAP, and (v) IMP response for different stimulation frequencies. If significant differences were found, pairwise comparisons were performed using nemenyi *post hoc* tests without any corrections to locate further differences. A Kruskal-Wallis test was used to detect differences between IMPD, EMD, and CMAP. Wilcoxon Matched pair tests were performed to locate further differences.

## Results

### Increasing Stimulation Intensity Level

**Figure 5:**
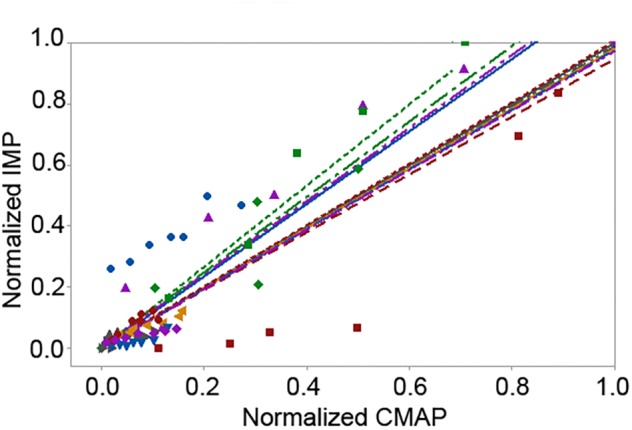
Scattered plot of the linear fit of the pooled normalized area of the intramuscular pressure (IMP) response with respect to the pooled normalized area of the compound muscle action potential (CMAP) levels up to the maximum CMAP. Different colors and shapes indicate different participants.

The peak IMP was significantly correlated with the amplitude of CMAP for each participant [*r* median (IQR) = 0.99 (0.05), *P* < 0.001]. The area of the IMP response was significantly correlated with the area of the CMAP as well [0.99 (0.18), *P* < 0.001] (Figure 5). The adjusted *r*-squared values based on multivariate regression were 93.8% and 93.4% for the amplitude and the area models, respectively. *Cook’s D* showed that none of the data points were identified as outliers.

**Figure 6:**
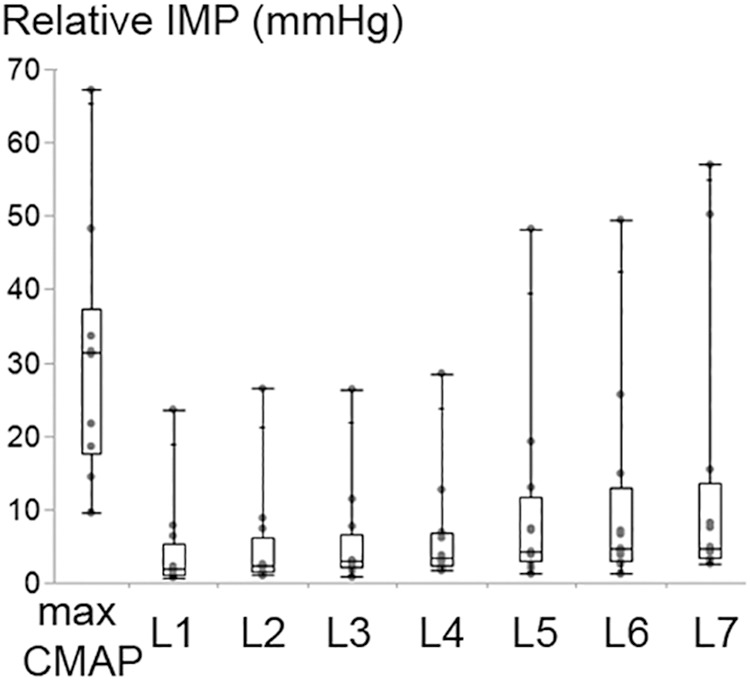
Box and whisker plot of intramuscular pressure (IMP) at different stimulation intensity levels. The stimulation level that generates maximum CMAP and level 1 (L1) refer to the maximum and minimum intensity applied, respectively. The IMP changed significantly with the increasing stimulation amplitude (*P* < 0.001).

Increasing the stimulation intensity resulted in a significant increase at the IMP response (*P* < 0.001) (Figure 6). Median (IQR) values for the max CMAP level, level 1 (L1), L2, L3, L4, L5, L6, and L7 equaled to 31.38 (19.75), 1.83 (4.33), 2.24 (4.62), 2.93 (4.49), 3.45 (4.39), 4.24 (8.75), 4.61 (9.90), 4.75 (10.15) mmHg, respectively. IMP responses at max CMAP and L1 were significantly different than the IMP responses at all other levels of activity (*P* < 0.001 for all pairwise comparisons). IMP responses at L2 were different than the IMP responses at L6 (*P* = 0.037) and L7 (*P* < 0.001). Additionally, the IMP measured at L7 was significantly higher than the IMP responses at L2 (*P* < 0.001) and L3 (*P* = 0.002).

### Stimulation Frequency

**Figure 7:**
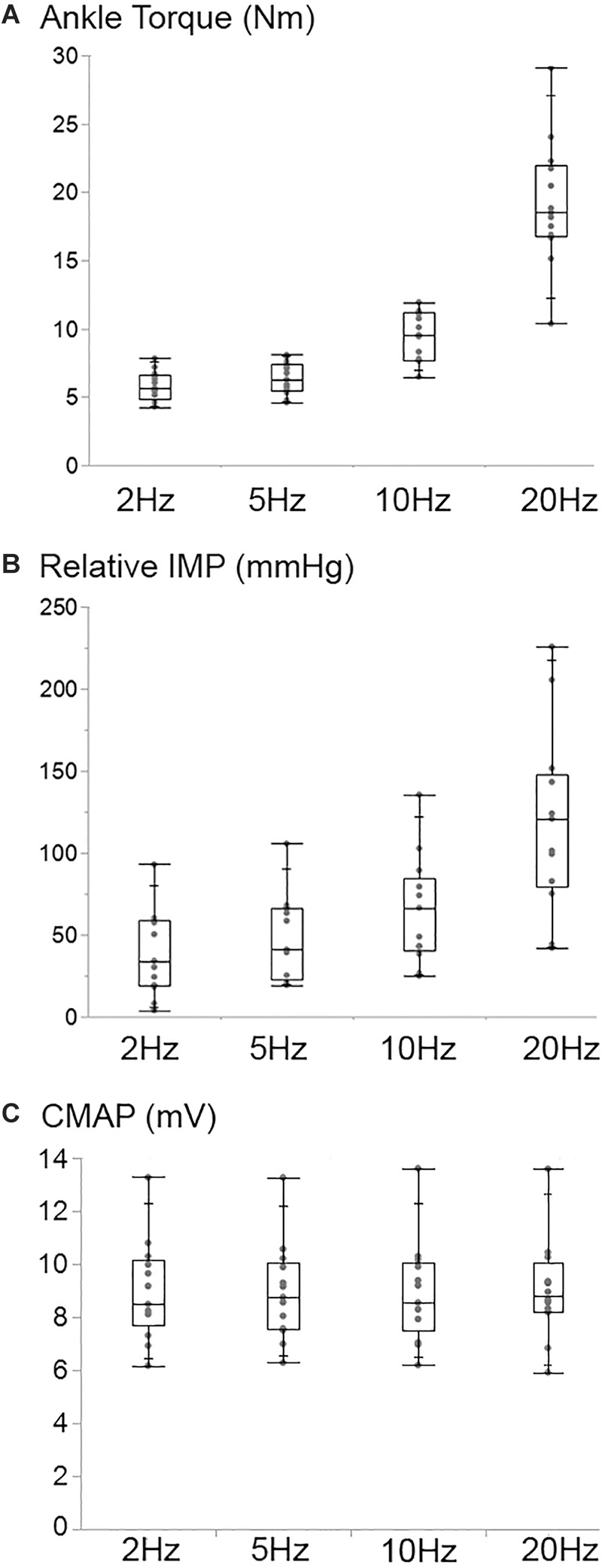
Box and whisker plot of the ankle torque **(A)**, the intramuscular pressure (Relative IMP) **(B)**, and the CMAP **(C)** values at different stimulation frequencies. The TA CMAP did not change (*P* = 0.809) whereas the ankle torque (*P* < 0.001) and the IMP (*P* < 0.001) increased significantly with the increasing stimulation frequency.

Increasing stimulation frequency did not change the CMAP values recorded from TA (*P* = 0.809) whereas it caused ankle torque (*P* < 0.001) as well as the peak IMP (*P* < 0.001) to change (Figure 7). *Post hoc* tests showed significant differences for ankle torque between each pair of stimulation levels except between the torques measured at 5 and 10 Hz (*P* = 0.071); and 10 and 20 Hz (*P* = 0.197) of stimulation. The ankle torque was higher during 20 Hz stimulation compared to 5 Hz (by 65.1% (9.8%), *P* < 0.001), and 2 Hz (by 68.6% (7.2%), *P* < 0.001). Similar to the change in torque, *post hoc* tests for the peak IMP showed significant differences between each pair of stimulations levels except between the IMP measured at 5 and 10 Hz (*P* = 0.197); and 10 and 20 Hz (*P* = 0.197) of stimulation. The peak IMP were higher during 20 Hz stimulation compared to 5 Hz [by 59.3% (16.4%), *P* < 0.001), and 2 Hz (by 68.2% (17.8%), *P* < 0.001].

T_IMPpeak_ [median (IQR) = 0.63 (0.41) s] was found to be significantly earlier than the T_Torquepeak_ [1.07 (0.58) s] if maximally stimulated (*P* = 0.003).

### Onset Delay

**Figure 8:**
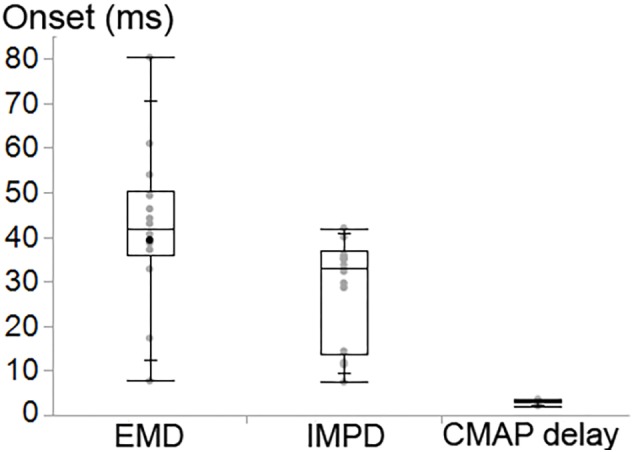
Box and whisker plot of the onset delay of the ankle torque (EMD), the intramuscular pressure (IMPD) and CMAP of tibialis anterior (TA).

The CMAP delay, EMD and IMPD were found to be significantly different (*P* < 0.001) (Figure 8). The ankle torque EMD [median (IQR) = 41.8 (14.4) ms] was later than the IMPD [33.0 (23.6) ms] as well as the CMAP delay [3.2 (0.6) ms].

## Discussion

The findings of the present study supported the hypotheses posed. The IMP is directly related to the amount of electrical activity of the muscle as measured by the TA CMAP. Increasing the CMAP by increasing the intensity of stimulation of the fibular nerve results in an IMP increase. Increasing the stimulation frequency on the other hand, increases the ankle torque as well as the TA IMP. The onset delay is the shortest for the electrical response (CMAP) and the longest for the mechanical response (torque). The novel finding is that the IMPD is earlier than the EMD. The results indicate that the methods used in this study allow us to quantify the electromechanical function of individual muscles rather than muscle groups. These findings may be useful in assessing patients with muscular disorders whose primary abnormality is a disorder of the contractile apparatus. Novel aspects of this method are discussed below.

### CMAP, IMP, and Clinical Relevance

The CMAP is a measure of the muscle fiber electrical activity after electrically stimulating the nerve innervating the muscle. The amplitude and the area of the CMAP is dependent on the number of motor axons; the number of muscle fibers innervated by each axon (motor unit), and the number of motor axons activated through electrical stimulation. A decrease of the CMAP can be a surrogate for nerve disorders such as neuropathy (Yuen et al., 1995; Rajabally et al., 2012), radiculopathy (Rubin and Whaley, 2010; Pawar et al., 2013), spinal muscular atrophy (Lewelt et al., 2010), or myopathy (Goodman et al., 2009; Szmidt-Salkowska et al., 2015; Kramer et al., 2018). It has been also shown that strength training improves the CMAP amplitude (Molin and Punga, 2016). However, in muscle disorders where the primary abnormality is only due to the contractile apparatus, like in early steroid myopathy, the measure of the electrical activity *per se* may not be sufficiently sensitive to detect an abnormality. In the present study, we analyzed the CMAPs as well as the IMP responses and found that both measures, the IMP amplitude and the area of response of IMP, are strongly correlated with those of CMAP. These findings suggest that IMP directly reflects the electrical activity of the TA muscle. To systematically test the IMP response for minimal increases in muscle activity, different levels of stimulation were applied starting from the lowest stimulation current that produced a recordable CMAP and then increased just by as much as it was necessary to produce an increase in the CMAP. This graded increase in current amplitude was an attempt to add the response of a single motor unit with each increment. However, McComas et al. (1971), who discussed the techniques for motor unit estimates (MUNE), outlined the difficulty of performing MUNE studies on large muscles like the TA. Therefore, we cannot claim that each increment is added by only one motor unit. If our assumption is correct, however, then the relative IMP increment per motor unit is calculated by using the differences between the stimulation levels and the IMP produced the average (SD) pressure that an electrically stimulated single motor unit generates would be 1.01 (0.42) mmHg. This needs to be validated by studying muscles where MUNE techniques are more reliable and reproducible. It should also be noted that the effects of inter-individual differences need to be considered while establishing an IMP as a measure of force. Nevertheless, our study suggests that measuring IMP has the potential to show force production capacity of motor units that will be helpful in assessing abnormal muscles.

Moreover, our data shows that IMP reflects the joint mechanical characteristics: Unlike the CMAP amplitude that does not further increase after supramaximal stimulation, IMP of TA and ankle torque increased with increasing stimulation frequency. Therefore, IMP of TA captures the change in joint torque generated by stimulation of the TA, something that is not possible to detect with CMAP measurements during electrical stimulation. This shows that while no change in the muscle contractility can be observed from CMAP, the local mechanics reflected by IMP and torque still change. IMP provides a unique opportunity to capture the mechanical changes by quantifying the hydrostatic pressure. This is consistent with a recent report showing that IMP reflects ankle torque during voluntary isometric contractions of TA (Ates et al., 2018b). During both voluntary contractions and nerve stimulation, quantifying force production capacity of muscles has clinical importance. Our previous and present findings indicate that IMP reflects electromechanical events associated with activated muscle and we suggest that IMP is a good candidate for monitoring muscle weakness.

Measuring torque could be an alternative approach but it is not easy to measure the response from one muscle and may not be sensitive enough to detect minimal changes in muscle function. Within a clinical setting, manual muscle testing is being used, however, it is subjective, depends on the skills of the examiner, and requires the full cooperation of the subject. IMP, on the other hand, is able to measure changes in the contractile apparatus in an individual muscle, the results are objective, and the results are independent of the effort of the subject. Measurements of IMP can be helpful in following the course of neuromuscular diseases, especially in treatment trials or studies of disease evolution. Additionally, previous studies showed that exercise promotes healthy aging (Cartee et al., 2016) and exercise planning is essential for older adults in particular (Bickel et al., 2011). Our findings suggest that collecting IMP can be used to follow the effects of aging and exercise dosing on muscle mechanics and musculoskeletal health.

It should be noted that any complaints about pain did not differ from that experienced with routine clinical needle electromyography performed by an experienced clinician.

### Electromechanical Delay and Its Implications

Presently, the EMD of ankle torque was assessed by measuring the onset of the ankle dorsiflexion torque generated by the TA activated via transcutaneous stimulation of the fibular nerve. To the best of our knowledge, there is no earlier report on the EMD of dorsiflexion torque generated by electrical stimulation. Recent reports show an ankle torque EMD of 111 ms (Ates et al., 2018b) or 140 to 310 ms of EMD depending on the frequency and the intensity of activity (Ubeda et al., 2017) measured during TA isometric voluntary contraction. However, EMD measured during voluntary contractions was shown to be more than twofold the EMD of plantar flexion torque measured during stimulated contractions (Hopkins et al., 2007). Stimulated contraction presumably does not follow the size principle of recruitment order where small motor units are activated first and larger fibers need to reach a threshold during voluntary activities (Henneman et al., 1965). Consequently, direct comparison of the EMD calculated during stimulation and voluntary contraction is not feasible. Considering the previous nerve stimulation studies, it appears that presently shown dorsiflexion torque EMD (42 ms) is slightly higher than the EMD of the ankle plantar flexion torque generated by (i) percutaneous stimulation of tibial nerve (9.7 ms) (Hopkins et al., 2007), and (ii) percutaneous stimulation of gastrocnemius muscle [11.6 ms (Nordez et al., 2009) or between 14.8 and 19.2 ms depending on ankle angle (Muraoka et al., 2004)]. Additionally, the EMD of elbow joint torque generated by stimulation of biceps brachii (BB) muscle ranged from 10.7 to 23.1 ms depending on the elbow joint angle (Hug et al., 2011; Sasaki et al., 2011; Lacourpaille et al., 2013a). Although some of the determinants of EMD such as (i) the method of calculation varying from using ultrafast ultrasound to EMG and (ii) the measurement conditions e.g., joint position and muscle slack length differ, these previous reports show consistently shorter onset delays for the gastrocnemius and BB compared to the present values for TA. One reason may be the architectural and functional differences between these muscles: TA is reported to be stiffer than BB and gastrocnemius at rest (Lacourpaille et al., 2012). This indicates a stiffer parallel and series elastic component determine the velocity of propagation of action potentials and muscle force transmission. BB as a fusiform muscle has an architectural advantage for force transmission. Both gastrocnemius and TA are pennate muscles, however, compared to the gastrocnemius (Narici et al., 1996), the TA muscle has longer fibers both at rest and during voluntary contractions (Simoneau-Buessinger et al., 2017). This may imply a different path for force transmission through the TA, where the properties of the parallel elastic component and the active part of the series elastic component of EMD are involved. More importantly, tendon length and stiffness are also major determinants of the EMD (Morl et al., 2012). TA muscle has a longer tendon which spans from lateral to the medial cuneiform and first metatarsal bones of the foot. This may delay the mechanical response as well as change the external measurement factors such as the transmission between foot and plate. Moreover, the stimulation method would also modify the EMD in part: In contrast to the present study where transcutaneous nerve stimulation was performed to generate CMAP, in all previous studies percutaneous stimulations were applied. Increasing the stimulation intensity would increase the number of motor units activated and extend the EMD (Lacourpaille et al., 2013b). Therefore, EMD measured during submaximal stimulations (e.g., Sasaki et al., 2011) might be shorter. Even if supramaximal stimulation is applied, all motor units may not be activated throughout the muscle. This may shorten the time of torque onset. Nevertheless, these studies showing large variation suggest that for longitudinal evaluations, measurement protocols need to be standardized.

IMPD is found to be earlier than EMD. This indicates that IMP reflects local intramuscular mechanical changes that occur before measured joint torque production. Recent studies showed that forces measured directly from the tendon do not necessarily represent the muscular condition for spastic muscles (Ates et al., 2013b, 2014, 2016; Yucesoy et al., 2017). Therefore, to better understand the musculoskeletal changes due to diseases, it is essential to obtain local properties of diseased muscles. IMP can provide that capability. The presently reported onset time of CMAP shows that the electrical response of muscle occurs the earliest after the stimulation. The onset delay information obtained from healthy adults in the present study can be used to detect and monitor the course of neuromuscular diseases such as myopathy and dystrophy that impair electro-mechanical transmission. Furthermore, the mechanical role of the fluid content of muscle, i.e., IMP phenomenon, was previously investigated under passive (Wheatley et al., 2017) and active (Jenkyn et al., 2002) conditions using a continuum mechanical model of rabbit TA muscle and finite element method. To better link the IMP to electrophysiological properties, continuum mechanical models have only limited predictive power. However, there are detailed biophysical multi-scale chemo-electromechanical skeletal muscle models (e.g., Rohrle et al., 2012) that capture, for instance, the electrophysiological characteristics of sarcomeres, include functional aspects of MU recruitment and predict EMG signals (Mordhorst et al., 2015) as a result of particular recruitment patterns. Implementation of IMP and EMD information into such skeletal muscle models would improve our understanding of muscle mechanics. Additionally, by this approach, these muscle models can be improved in order to predict muscle weakness, e.g., myopathy related changes, and the effects of therapies.

### *In vivo* Muscle Positioning, IMP Response, and Future Directions

Although the TA is a superficial muscle, it is surrounded by the tibia and other anterior crural muscles. Contraction of muscles results in shape changes that increase the intermuscular pressure between neighboring muscles (Reinhardt et al., 2016). This may cause an additional increase in IMP. Results on isolated rat muscles (Siebert et al., 2014) as well as on human calf muscles (Siebert et al., 2018) have shown that muscle compression influences muscle force production capacity. It was recently argued that IMP (Ates et al., 2018b), the resultant forces at muscle tendons (Maas et al., 2003; Ates et al., 2013a) as well as the stiffness of muscles (Ates et al., 2018a) are determined not only by the architecture of an individual muscle but also the relative positioning of the neighboring muscles. For example, to be able to apply the IMP method to deeper muscles, which are almost completely surrounded by neighboring muscles, the effects of connective tissues, and bones, on a muscles’ *in vivo* positioning and on the IMP response should be investigated.

When maximally stimulated, we found that the IMP reaches to its peak value within 0.63 s. This is almost 50% earlier than the T_Torquepeak_. Consistent with its onset time being earlier, as a local measure, IMP peaks faster. In contrast, ankle torque shows a slower increase and generates an apparent plateau. We also observed that IMP level drops soon after generating the peak value. This has occurred at high frequency stimulations. This seems to be related to either rapid redistribution of interstitial fluids within the muscle causing the local pressure to drop or viscoelastic muscle properties. Since it represents the acute condition, we chose to analyze the peak values in the present study. However, the relationship between intramuscular pressure and viscoelastic properties of muscles at different activity conditions needs to be further investigated with additional studies, e.g., in animal muscles. Furthermore, we observed pressure drops to negative values (around -10 mmHg) after high levels of activation (in particular at the end of electrical stimulation). In contrast, IMP output does not necessarily show negative values after it voluntarily contracts, i.e., a participant returns to the resting state slowly. The rapid drops in pressure at the end of electrical stimulation might be due to the quick bend of diaphragm of the sensor to the other side. This might have occurred due to the movement of sensor itself despite the fact that the IMP sensor has an anchor at the tip and attaches to a group of muscle fibers. As it remains attached, the IMP sensor moves together with the specific muscle fiber group that it attached to, e.g., moves during muscle contraction. Therefore, slip or movement of the sensor is not expected. For each subject, this has been checked and confirmed before data acquisition whether the tip of sensor is anchored using ultrasound as well as observing the IMP signal output at low muscle contractions.

## Conclusion

The findings of the present study indicate that IMP reflects *in vivo* TA muscle activity during electrical stimulation. IMP is correlated with CMAP: it increases with the increasing muscular activity imposed by nerve stimulation. Even though they show related but distinct response patterns, IMP reflected the increase in ankle torque during various stimulation frequencies. Therefore, we conclude that IMP represents muscle active electromechanical characteristics. The earlier IMPD indicates that IMP represents local mechanical response. Our findings suggest that IMP can be designed to serve as a minimally-invasive tool to evaluate the electromechanical performance of individual muscles e.g., to detect muscle weakness.

## Author Contributions

All the authors contributed to the development of the project, experimental preparations, and data collection. FA performed data analyzes and manuscript writing. KK contributed to editing and critical appraisal of the manuscript. All the authors approved the final submitted manuscript.

## Conflict of Interest Statement

The authors declare that the research was conducted in the absence of any commercial or financial relationships that could be construed as a potential conflict of interest.
